# Integrated Care in the Era of COVID-19: Turning Vision Into Reality With Digital Health

**DOI:** 10.3389/fdgth.2021.647938

**Published:** 2021-08-03

**Authors:** Angelina Kouroubali, Haridimos Kondylakis, Dimitrios G. Katehakis

**Affiliations:** Institute of Computer Science, Foundation for Research and Technology-Hellas (FORTH-ICS), Heraklion, Greece

**Keywords:** integrated care, interoperability, digital health, COVID-19, FHIR, eHealth, EHR

## Abstract

The lives of millions of people have been affected during the coronavirus pandemic that spread throughout the world in 2020. Society is changing establishing new norms for healthcare education, social life, and business. Digital health has seen an accelerated implementation throughout the world in response to the pandemic challenges. In this perspective paper, the authors highlight the features that digital platforms are important to have in order to support integrated care during a pandemic. The features of the digital platform *Safe in COVID-19* are used as an example. Integrated care can only be supported when healthcare data is available and can be sharable and reusable. Healthcare data is essential to support effective prevention, prediction, and disease management. Data available in personal health apps can be sharable and reusable when apps follow interoperability guidelines for semantics and data management. The authors also highlight that not only technical but also political and social barriers need to be addressed in order to achieve integrated care in practice.

## Introduction

The coronavirus disease 2019 (COVID-19) has caused a worldwide pandemic (1), that has disrupted the lives of millions of people across the globe (2). Confinement measures have focused on the reduction of the spread of the epidemic and the minimization of the load of morbidity and mortality so that healthcare systems remain functional (3). Healthcare measures have focused on treatment, case isolation and contact tracing. Many digital tools have been developed to assist with contact tracing and case identification (4). Despite the fact that it is mobile solutions that enable the automatic detection of contacts, saving precious hours of work of public health staff, still several concerns have been raised from the very beginning in regards to legal and technical safeguards (5). In addition, several solutions have been built in isolation without taking into consideration interoperability specifications for rendering the collected data sharable and reusable. Interoperable systems can make data sharable and reusable introducing many opportunities for growth and improvement. Eliminating data silos and automating data integration supports the recognition of unseen patterns, offers opportunities to apply new intelligence to service patients and care-givers, creating value across the care continuum (6).

Due to the burden of multi-morbidity, which is growing in the European countries, because of the aging population, an increasing number of people find themselves in having to deal with more than one chronic or long-term conditions (7–10). This brings the need for the provision of integrated care (11, 12), in order for people to receive appropriate treatment across the continuum of healthcare delivery, including rapid response in cases of a pandemic break that may affect disproportionally heavy patients with certain chronic conditions.

This paper presents the authors' perspective on how digital technologies can help make integrated care a reality addressing the needs of governments to efficiently respond to pandemics such as COVID-19. It elaborates on the requirements necessary so that digital platforms can effectually act as outbreak response tools. These include appropriate and effective data management and semantics as well as interoperable personal health apps that address the needs of citizens to effectively support contact tracing while staying safe in the pandemic. In addition, the authors highlight the existing technological, political and social barriers that need to be overcome for the effective digital management of the pandemic.

In order to illustrate this perspective, the authors take as an example *Safe in COVID-19* (13), a digital platform designed to address the need for sharing information between patients and physicians as well as providing data for public health authorities. The architecture of the platform has already been published in Kouroubali et al. (13), and is based upon existing work on personal health record systems (14, 15) and the development of integrated care solutions to effectively support personal health management and public health (16). We present the modules that such an app should employee for the effective management of COVID-19 and we elaborate on its effective promotion through the available political and social channels, presenting also the many potential benefits.

## Digital Health In Covid-19

Mobile technologies have been widely developed as a response to the pandemic with a variety of features (17). There are technologies that facilitate self-assessment at home, training, information sharing, contact tracing, and decision making, swiftly offering effective and usable tools against the COVID-19 pandemic. Some examples include platforms, like COVIDSafe in Australia (https://www.health.gov.au/resources/apps-and-tools/covidsafe-app) that offer the ability to document registered isolation and to allow public health to conduct appropriate analysis and research; COVID Symptom Tracker (https://covid.joinzoe.com/us) in the US, that tracks the onset and progression of symptoms to pinpoint virus hot spots and to help slow the spread of the disease; MaladieCoronavirus (https://maladiecoronavirus.fr/) in France for symptom checking and self management; Corona-Warn-App (https://www.coronawarn.app/en/) for tracing the spread and providing information; Covid19.es (https://covid19.es/) in Spain for symptom checking and tracing the spread; Helsenorge (https://www.helsenorge.no/) for symptom checking, tracing and information providing; and the software application Go.Data (https://www.who.int/godata), developed by the Global Outbreak Alert and Response Network (GOARN) to manage case-contact relationships and the follow-up of contacts. Go.data has been deployed to over 35 countries in support of the COVID-19 Pandemic response. In addition, several countries, such as France, Croatia, Germany, and Norway have already developed e-government apps for their citizens, under the direction or with the endorsement of public organizations (18).

A systematic survey (17) has shown that mobile apps can benefit citizens, health professionals and decision makers in facing the COVID-19 pandemic. The challenges that these apps have addressed, include increasing the reach of reliable information to both citizens and health professionals as well as reducing misinformation and confusion. App features include symptom tracking, mental health support, and home-monitoring. Additionally, the information collected in the mobile apps can help discover new predictors about the course of the disease, optimize healthcare resource allocation and reduce the burden to hospitals (17).

Although massive numbers of data are collected in digital health systems, they exist in isolated data islands that are not widely available and hence cannot be used to enable further statistical analysis and prediction making. Patient data, even when available, they cannot be readily used by mobile apps. Data structure and form are often not appropriate for data exchange. The COVID-19 pandemic has created an even greater need for healthcare professionals to gain knowledge about how their patients' health is evolving in real time. Also, decision makers need to be able to monitor citizens who report COVID-19 related symptoms but have not been tested with a diagnostic test for COVID-19.

Implementing remote care monitoring is likely to generate long-term benefits and help with the healthcare challenges as part of the national healthcare system. Despite the potential benefits of digital solutions, their adoption has been slow. Digital solution developers posit technical and privacy issues as the main reasons for the slow adoption of digital solutions (17). Non-functional specifications essential for the delivery of trustworthy apps are relevant to compliance with GDPR provisions, role-based access to patient depending on end-user roles, ensuring accuracy and security of the data, the ability to communicate with other applications and registries using international standards (e.g., COVID-19 registries, nationally approved terminologies, citizen and healthcare professional identification services, etc.), as well as compliance with approved medical protocols.

In addition, a full range of features is not included in most solutions (19). An important need includes the development and utilization of decision support tools to assist policy optimization for minimizing negative socio-economic impact, while contributing to the containment of the pandemic. Furthermore, the pandemic has revealed preparedness shortcomings in public health (3). Social, organizational, and technological factors need to be addressed in order to allow the adoption of these eHealth tools. Further steps will include the involvement of patients to further elaborate requirements for usable and meaningful solutions. Uptake of the mobile eHealth resources will require a significant change in management efforts and the redesign of existing models of care (20–22).

It is important to note that even though digital solutions for remote care can be a valuable alternative to the isolation conditions imposed during a pandemic crisis, they cannot substitute face to face physical examinations which include human contact and communications (23). In addition, financial and legal constrains in technology readiness of healthcare systems can also act as a barrier to significant and long-term digital transformation, despite the rare opportunity of the current crisis (24). Integration with third-party applications would enable the use of the platform as an extension of the already existing hospital information systems enhancing the digital management of COVID-19 cases (25–27).

*Safe in COVID-19* digital platform intends to respond to the challenges leveraging on existing experience and robust implemented technologies. It is built on an extensible architecture focusing on the needs of all involved stakeholders including citizens and their families, healthcare professionals and public authorities. The platform addresses user information and communication needs (13). Data is collected and stored in such a way so that semantic and big data technologies can utilize them to enable, decision support and appropriate proximity tracking when needed. Further details are provided in the next section whereas Table 1 summarizes the differences of some of the available applications in the area, demonstrating the advantages of the Safe in COVID-19.

**Table 1:** A comparison of the features of key platforms for COVID-19.

	**Country**	**Symptom checker and self diagnosis**	**Tracing the spread**	**Providing information**	**Home support for self-management**	**Communication with medical staff**	**AI enabled**	**Interoperable**
Safe in COVID-19	Greece	x		x	x	x	x	x
COVIDsafe	Australia		x	x				
COVID symptom tracker	US	x	x					
Maladie coronavirus	France	x			x			
Corona-Warn-App	Germany		x	x				
Helsenorge	Norway	x	x	x				
COVID-19.es	Spain	x	x		x			
Go. Data	More than 35 countries		x					

## Safe In Covid-19 Digital Platform

*Safe in COVID-19* is an expandable digital platform that was developed in an effort to support national authorities in Greece. The platform provides tools for public authorities, healthcare professionals, citizens and their families offering an integrated care perspective to the ongoing pandemic.

**Figure 1:**
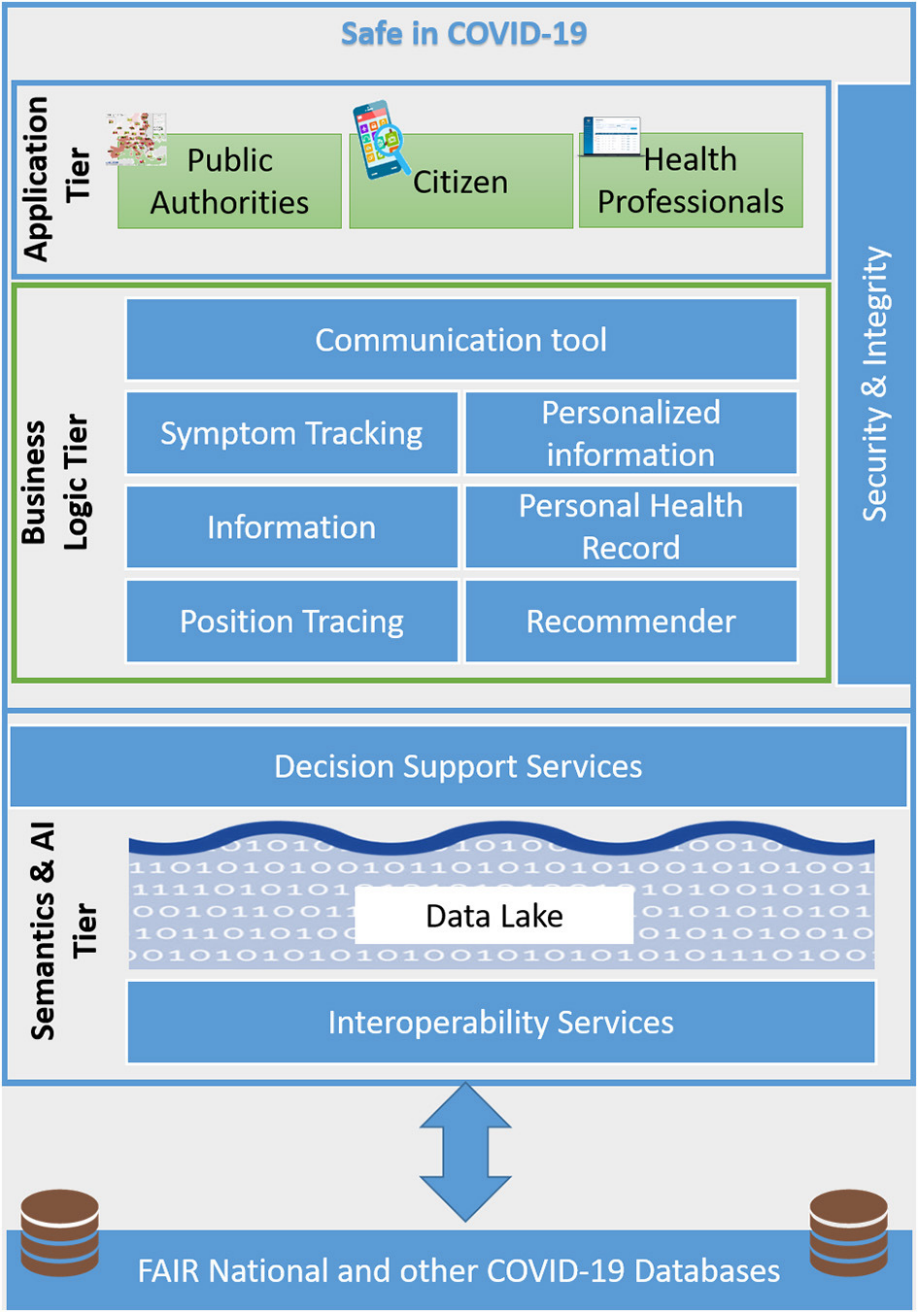
Platform architecture.

The platform architecture is shown in Figure 1 and consists of the Application Tier providing the front-end applications to the end-users, and the Business Logic Tier offering the intelligent functionality (13). All these layers are supported by security and integrity services as described below.

### Application Tier

The *Safe in COVID-19* solution includes the following applications: (i) a web application for public health authorities to give a complete picture of the status regarding the spread of the disease at a national level and the measures taken by healthcare services; (ii) a web application for healthcare professionals that supports online communication with registered patients in order to provide personalized information and coaching and instant access to patient reported symptoms related to COVID-19 disease for monitoring their health status; and (iii) a mobile application (Android/iOS) for citizens that allows recording on a daily basis their health status and communicate synchronously or asynchronously with healthcare professionals in order to receive personalized instructions for managing their health. Certainly, web apps could be used instead of using smart phones for the citizens. However, we believe that packing the app as a mobile app makes it easier for users even without appropriate digital skills to navigate and enjoys additional integration with their mobile phone (push messages etc.).

### Business Logic Tier

This tier includes the necessary services for handling the logic behind the graphical user interface. More specifically as the *Safe in COVID-19* framework is based on and significantly extends a fully-fledged, state of the art PHR system, it includes the personal health records and user profiling out of the box. However, specific modules have been customized for COVID-19. The symptom module has been extended to include specific symptoms of COVID-19, and the communication module has been extended to enable continuous communication with the healthcare team. Other modules such as the position tracing and the recommendations have been implemented specifically for addressing the COVID-19 outbreak. All collected data are stored in the data lake, enabling visual exploration through the application tier. Different levels of care and health services are brought together through online interaction with national digital health portals that provide secure application programming interfaces for patient, healthcare professional and insurance organizations applications. Typical services include access to national EHR services, ePrescription and national registries with COVID-19 patients.

### Security and Integrity

Handling personal health data requires specific attention to the security and the integrity of the collected data. As such, the *Safe in COVID-19* framework does not store specific patient identification data, but only unique codes generated randomly by the system and shared with the health authorities. In addition, those codes can also be generated by the health authorities for the confirmed COVID-19 cases. Data processing has been minimized only for the persons who need it whereas, all data is encrypted in order to further increase security and privacy. Further, every data access is recorded, whereas the necessary consent is also granted by the citizens with full information about the intended processing of data. In order to verify legitimate users (i.e., entering on purpose false positives, undermining this way faith in the system) users may need to receive appropriate codes when entering data into the app to be confirmed by the server. Position tracking is legitimized by relying on a voluntary adoption by the users for each of the intended purposes.

### Semantics and AI Tier

Further, the *Safe in COVID-19* framework goes beyond simple data dashboards, by providing intelligent dashboards that enable the multidimensional exploration and filtering of the available data. Internally we have (a) external data directly ingested in the data lake (b) the database of the adapted PHR-C (the schema is based on the Open EHR). All data are mapped to an ontology, which enables the semantic uplifting of the available data and their homogenization and integration. The ontology currently used is an extension of the MHA semantic core ontology (28), a high-level ontology integrating several standard taxonomies and terminologies used for modeling health data. We have extended it with just minor additions to completely cover our needs for COVID-19. Exploiting the semantic meaning of the data machine learning algorithms and epidemiological models can be incorporated in order to make predictions on virus spread on a specific geographic region, combining the available data with external regional, country and health-system data that might be available. Being able to predict a few days in advance the number of beds needed is essential for health organizations to have time to organize resources to meet the health needs of their reference population (outsourcing of services, staffing, hotel sanitation, purchase of respirators, etc.). We have to note that all modules in the business logic tier access a homogeneized, semantically uplifted and cleaned version of the available data staged in the data lake. However, we also maintain the raw staged data as we wouldn't like to lose any important information through errors in data cleaning and we would like to be able to repeat the cleaning steps if needed.

## Digital Platforms As Outbreak Response Tools

Establishing a close cooperation with public authorities is imperative in order to put the necessary applications into operation, since public health falls under their jurisdiction. Specific details about the development and deployment of COVID-19 relevant solution at a national level can only be established through compliance with approved medical protocols, interoperability with national registries for citizen identification and COVID-19, quality assurance, and the existence of the appropriate legal framework. Digital platforms can be effective when several interrelated factors are in place such as: (i) a public health system with a comprehensive national epidemiologic strategy (ii) a technology and architecture model that promotes interoperability for data sharing, and data re-use; (iii) widespread connection with mobile devices, and (iv) the appropriate regulatory and legislative conditions for the use of integrated digital solutions for all stakeholders safeguarding safety and privacy for all.

To leverage the individual benefits of mobile apps and other digital technologies it is important to have an interoperability layer to enable the various such solutions to exchange data. A unifying layer will facilitate the exchange of meaningful data in the appropriate format and needs to be implemented on top of the individual proposed solutions. All collected data should be made immediately findable and accessible through fully anonymization and publishing on the web using digital object identifiers (DOIs) and other permanent identifiers. This process is called data FAIRification.

Relevant ontologies, such as the MHA semantic core ontology (28), and other and approaches can be used for semantically uplifting available data through mapping and/ or annotating using ontology terms. This allows the rapid re-use of the available data, enabling a common understanding and offering a rich set of terms for documenting and adding meta-data to the data provided by the platform. Appropriately homogenizing and integrating both app-specific data with external data from regional, country and health-system data sources can leverage and combine available knowledge for artificial intelligence models, enabling better and more useful predictions. Multiple epidemiological models are currently being developed for COVID-19 and could exploit the available data to make predictions on the disease spread further guiding healthcare services and personnel allocation. On the other hand, early patient identification through machine learning and the incorporation of risk models in such apps has a great potential for better helping those in higher risks.

The use of fast healthcare interoperability resources (FHIR) can enable and facilitate interoperability with existing hospital health systems. FHIR should be used for the representation of the medical data related to COVID-19 (29). COVID-19 Patient Reported Outcome Observations (30) have already been established including symptoms such as cough, fatigue, pain in throat, dyspnea, headache, diarrhea, nausea, loss of sense of smell, and temperature. FHIR resources such as Problem and Condition, are also appropriate for representation of the underlying diseases related to COVID-19. These resources can be automatically published and data properly anonymized through a FHIR Server. Any hospital information system may act as a data source for the *Safe in COVID-19* FHIR architecture, since they can publish patient laboratory results for COVID-19 tests into the FHIR Server. Available applications then will be able retrieve the test results from the FHIR server and present them to the end users.

Privacy and trust are key concerns for individuals considering the adoption of personal health systems. As data are expected to be directly shared upon their creation appropriate anonymization techniques should be enforced for sharing information among patients, and their relatives, doctors, researchers, policy makers and governments. Digital solutions need to provide appropriate explanations to citizens, highlighting the importance of sharing health information for “flattening the curve” and explaining all measures taken to ensure anonymity and confidentiality.

Extending existing digital services with novel solutions can increase public value. Efficiency and effectiveness are facilitated through the use of wider range of available tools such as the provision of legitimate user orientation, high level of participation, legality, and equity. Responses to COVID-19 have largely not been integrated, leading to adverse outcomes. Innovative digital solutions can exhibit their highest impact when they integrate and interoperate with existing healthcare services and infrastructures.

## Discussion And Conclusion

Digital platforms can act as disease outbreak support tools when they are able to integrate within existing digital health services. This paper presented the overall requirements and conditions that are essential in order to develop digital platforms that can be useful to all healthcare stakeholders including public health authorities, healthcare professionals and citizens (31). The *Safe in COVID-19* platform was used to illustrate in practice what the healthcare ecosystem can gain form an integrated innovative digital service, demonstrating the components that should be available and the challenges for adopting them in practice.

Providing the means to securely link reliable data has the potential to support care anywhere at any time. When coupled with advanced analysis, then digital systems can be transformed into smart systems to better support integrated care. Of course, relevant challenges linked to organizational, financial, legal and interoperability issues will always need to be properly addressed at different configuration settings.

Integrating novel digital solutions can provide opportunities to support and strengthen health systems in the diagnosis, treatment, monitoring, citizen empowerment through information, public health surveillance and epidemiology (4, 12, 32).

Foreseen benefits for public health authorities include decongestion of healthcare units, provision of real-time information on the evolution of suspected, candidate and confirmed cases, online monitoring of the spread of the virus, and effective decision-making regarding required measures. Benefits to healthcare professionals include support in managing patients, reduced time for direct contact with patients, more efficient case management, and improved working conditions. Benefits for citizens include systematic recording of symptoms, self-assessment, access to personalized information, and instructions and reminders based on their overall health status.

*Safe in COVID-19* offers an example of an integrated platform offering multiple benefits for the users in alignment with relevant national eHealth initiatives. It can operate efficiently under the appropriate regional or national infrastructures with links to medical and diagnostic centers around the country and the size of the population under consideration. Such platforms can support the return to the “new normal” with less stress and more security for individuals, more direct and safer management of patients by physicians, and better possibilities for monitoring the epidemic by public health authorities.

The healthcare systems can act as drivers of change and facilitate the adoption of similar eHealth technologies based on need. As technology adoption barriers have decreased due to the current pandemic (33), digital transformation in healthcare can accelerate the use of novel technologies. eHealth solutions can be implemented rapidly and can offer essential tools in supporting the COVID-19 pandemic for all stakeholders including citizens, healthcare providers, policy makers and governments, promoting coordination to ensure appropriate management of this crisis. Future research could focus on the assessment of the cost-effectiveness of digital health apps for the management of COVID-19 with the introduction of key performance indicators.

Digital solutions can help support integrated care in the era of COVID-19, on a larger scale. Healthcare systems need to focus on flexibility and interoperability to achieve sustainability and to realize the vision for healthcare at the point of need. Turning vision into reality requires taking the responsibility to act now to ensure health systems and people continue to benefit.

## Data Availability Statement

The original contributions presented in the study are included in the article/supplementary material, further inquiries can be directed to the corresponding author/s.

## Author Contributions

All authors listed have made a substantial, direct and intellectual contribution to the work, and approved it for publication.

## Conflict of Interest

The authors declare that the research was conducted in the absence of any commercial or financial relationships that could be construed as a potential conflict of interest.

## Publisher's Note

All claims expressed in this article are solely those of the authors and do not necessarily represent those of their affiliated organizations, or those of the publisher, the editors and the reviewers. Any product that may be evaluated in this article, or claim that may be made by its manufacturer, is not guaranteed or endorsed by the publisher.
